# Making microbial genomics work for clinical and public health microbiology

**DOI:** 10.1099/mgen.0.000900

**Published:** 2022-09-16

**Authors:** Taj Azarian, Norelle L. Sherry, Kate Baker, Kathryn E. Holt, Iruka N. Okeke

**Affiliations:** ^1^​ Burnett School of Biomedical Sciences, University of Central Florida, Orlando, FL, USA; ^2^​ Microbiological Diagnostic Unit Public Health Laboratory, Department of Microbiology and Immunology, University of Melbourne at the Peter Doherty Institute for Infection and Immunity, Melbourne, Victoria, Australia; ^3^​ Department of Clinical Infection, Microbiology and Immunology, Institute of Infection, Veterinary and Ecological Sciences, University of Liverpool, Liverpool, UK; ^4^​ Department of Infection Biology, Faculty of Infectious and Tropical Diseases, London School of Hygiene & Tropical Medicine, London WC1E 7HT, UK; ^5^​ Department of Infectious Diseases, Central Clinical School, Monash University, Melbourne, Victoria 3004, Australia; ^6^​ Department of Pharmaceutical Microbiology, Faculty of Pharmacy, University of Ibadan, Ibadan, Nigeria

## Full-Text

The advent of low-cost pathogen genome sequencing amenable to routine use has considerable implications for the future of infectious disease diagnostics and may be the most impactful development for public health surveillance and investigation in the 21st century. In particular, it has brought unprecedented precision to core tasks such as species identification, sub-species-level typing and outbreak investigation. Recent examples of pathogen genomics implemented in public health reference laboratories, published in *Microbial Genomics*, include national surveillance of influenza [1], SARS-CoV-2 [2], typhoidal *Salmonella* [3] and *Neisseria gonorrhoeae* [4], and outbreak investigation in a hospital critical care unit [5]. These examples notwithstanding, there remain significant barriers to full implementation of sequencing technology in clinical and public health settings laboratories. As the idiom alludes, *the devil is in the detail,* and the workflows needed to generate, analyse and interpret sequence data reliably in validated frameworks to guide clinical or public health practice are complex and multi-faceted. Understanding them scientifically, through research, is a prerequisite to confidently and sustainably apply genomics beyond academic frameworks, especially in the delivery of health care and public health [6].

There has been a rapid increase in pathogen sequencing capacity facilitated by considerable infrastructure investment during the global response to the SARS-CoV-2 pandemic. As a result, there is an opportunity to utilize the momentum generated by recent efforts to accelerate progress toward broader implementation. Toward this goal, *Microbial Genomics* is pleased to launch this special collection on the ‘Implementation of Genomics in Clinical and Public Health Microbiology’. Here we highlight recent *Microbial Genomics* articles on this theme that will be included in the Implementation Collection, and invite new submissions that further elucidate the remaining steps towards implementation and exemplify the utility of pathogen genomics in clinical and public health practice.

The past decade has seen a proliferation of innovative method development for applied microbial genomics, spanning wet-lab protocols for nucleic acid extraction and library preparation to bioinformatics pipelines and data visualization. Many such articles number among the most cited papers in *Microbial Genomics*. Recent examples, included in the Implementation Collection, address time-critical bacterial pathogen characterization [7], rapid investigation of antibiotic-resistant bacteria [8] and strain-level metagenomics for outbreak investigation [9].

However, implementation of pathogen genomics into routine clinical or public health practice requires more than simply choosing a protocol and software package. Thoughtful consideration must be given to (i) validation, both internal and external – does the method yield accurate answers (and to what degree)? compared to which standard? to what extent does this translate across health systems?; (ii) benchmarking of performance – accuracy, precision and efficiency compared to alternatives; (iii) external quality assessment – do we obtain consistent results across labs applying the same method on the same samples? do the same interpretive criteria apply across settings?; (iv) results reporting and harmonization – can results be compared with those we have generated previously or that other labs are generating? is the turnaround time for reliable results short enough to allow for a useful clinical or public health response? are the results able to be accurately interpreted by the recipient, providing actionable data?; (v) cost effectiveness – can the method deliver useful results at a price that is justifiable within health systems and public health budgets?; (vi) data sharing – how can sequencing data and source information be shared for broader public benefit, while balancing consequence, equity and privacy with timeliness and utility?; and (vii) evaluation **–** what are the outcomes we are looking for, and how do we measure the success of implementation? For authors embarking on new studies, we recommend following the recently published framework for evaluating whole-genome sequencing in public health.

Advancement in these areas is further complicated because genomic data yield higher granularity information from existing ‘gold standard’ methods which is sometimes discrepant, making it difficult to decide on benchmarks for comparing new approaches and methods – whether this be for species identification, detection of antimicrobial resistance determinants or outbreak detection. After all, the goal of implementing genomics is not just to replace older methods, but to enhance our overall ability to comprehend emergent clinical and public health problems and thereby inform more effective action. Yet demonstrating that a particular approach leads to better outcomes is enormously challenging, especially when the desired outcome is prevention of something that would otherwise have occurred, such as an ongoing outbreak. Hence while ‘validation’ and ‘evaluation’ may sound dull, the reality is that much creativity and innovation is needed in this space, and we hope to stimulate and promote such activity with this Implementation Collection.

To support the goal of widespread implementation, we believe there is also a specific need for more multi-centre studies, generating consensus around technical and process questions that can apply across settings, with evaluation of both health and economic outcomes. Recent articles published in *Microbial Genomics* that embody this theme, and are included in the Implementation Collection, include:

- ‘Discordant bioinformatic predictions of antimicrobial resistance from whole-genome sequencing data of bacterial isolates: an inter-laboratory study’ [10]
- ‘Multi-laboratory evaluation of the Illumina iSeq platform for whole genome sequencing of *Salmonella *, *Escherichia coli* and *Listeria *’ [11]
- ‘Centre-specific bacterial pathogen typing affects infection-control decision making’ [12]
- ‘Harmonization of whole-genome sequencing for outbreak surveillance of *Enterobacteriaceae* and *Enterococci*’ [13]
- ‘Validation strategy of a bioinformatics whole genome sequencing workflow for Shiga toxin-producing *Escherichia coli* using a reference collection extensively characterized with conventional methods’ [14]
- ‘GenomeTrakr proficiency testing for foodborne pathogen surveillance: An exercise from 2015’ [15]

We encourage new submissions to the Implementation Collection that present the results of inter-laboratory evaluation of genomics methods, tools and databases; frameworks for implementation and training; articles evaluating genomics methods against established standards; and proposing standards, proficiency tests or reporting proforma for applied microbial genomics. We also welcome articles proposing and/or evaluating new genomics-based methodologies for clinical or public health microbiology (including bacteriology, virology, parasitology and mycology). For authors embarking on new studies, we recommend following the recently published framework for evaluating whole-genome sequencing in public health [16].

Additionally, we note that there is a significant need for better data linkage, visualization and decision-support tools for genomics implementation. Such tools can provide clinical and public health practitioners with contextualizing and co-visualizing genomic data alongside epidemiological and clinical data to ultimately support confident decision-making. We encourage research and development in this area, and warmly welcome submissions introducing and evaluating such tools for inclusion in the Implementation Collection.

We are eager to receive your innovative and novel research for consideration. Together with invited commentaries we aim to curate a collection with broad interest to researchers and practitioners in microbial genomics and clinical and public health practice. These articles will hopefully guide the next 5 years in our field as we move toward the realization of widespread routine pathogen genomics.
